# A handheld UV-C light-emitting diode decreases environmental contamination near the operative field

**DOI:** 10.1017/ash.2024.409

**Published:** 2025-01-06

**Authors:** Rachael A. Turner, Roseann M. Johnson, Yasmin Yazdani-Farsad, Jessell Owens, Douglas A. Dennis, Jason M. Jennings

**Affiliations:** 1 University of Texas Rio Grande Valley School of Medicine, Edinburg, TX, USA; 2 Colorado Joint Replacement, Denver, CO, USA; 3 Great Basin Orthopaedics, Reno, NV, USA; 4 Department of Mechanical and Materials Engineering, University of Denver, Denver, CO, USA; 5 Department of Orthopaedics, University of Colorado School of Medicine, Denver, CO, USA; 6 Department of Biomedical Engineering, University of Tennessee, Knoxville, TN, USA

## Abstract

**Introduction:**

Periprosthetic joint infection (PJI) may result from pathogen-to-patient transmission within the environment. High-touch surfaces (HTS) areas near the operative field from previous studies had been identified as the least likely to be thoroughly cleaned between operative cases and were utilized for this study. The purpose of this study was to assess the impact of a handheld ultraviolet-c (UV-C) light-emitting diode (LED) disinfection device on the decontamination of HTS in the operating room.

**Methods:**

This prospective study was conducted between 03/02/2021 and 04/20/2021. Tryptic soy agar contact plates were used to determine the bacterial load of the selected surfaces before the initiation of the case, after the case was complete, before manual cleaning, and after disinfection of the LED device. The plates were then incubated for 48 hours at 36º +/–1° C. Colony forming units (CFU) were recorded 48 hours after incubation. Mean, median, and range of CFU were recorded.

**Results:**

Average CFU per surface before and after the surgical case were 14.1 (range 0–200) and 13.5 (range 0–200) respectively, these were not significantly different (*P* = 0.9397). Manual cleaning reduced average CFU by 74% to 3.35 (range 0–200) per surface (*P* = 0.0162). Disinfection with the handheld LED unit further reduced the average CFU by 92% to 0.28 (range 0–4) per surface (*P* < 0.0001).

**Conclusions:**

A handheld UV-C LED disinfection device may decrease environmental contamination near the operative field in HTS areas. Further research is warranted with this technology to determine if this correlates with a decrease in PJI.

## Introduction

The number of total joint arthroplasty (TJA) procedures has been increasing along with the total number of surgical site and periprosthetic joint infections (PJI).^1^ PJI is difficult to diagnose and is one of the most catastrophic complications of TJA associated with increased morbidity and substantial cost.^2^ The development of PJI includes multiple risk factors associated with the patient and environment;^2^ thus, prevention of PJI is multifactorial.^3,4^ Environmental transmission of pathogen to patient is a potential cause of contamination causing PJI, and there is a potential relationship between the number of colony forming units (CFUs) in the operative environment and the incidence of PJI.^5^

High-touch surfaces (HTS) in the operating room (OR) at risk for gaps in cleaning have been identified.^7–9^ These studies have suggested many surfaces (i.e. anesthesia cart, nurses station, OR bed) have not been cleaned thoroughly which has led to recommendations for improvements in targeted cleaning and staff education. There is growing evidence that the hospital environment, including the OR, is often not cleaned thoroughly or in a manner consistent with relevant hospital policies.^7,10^ These deficiencies have targeted visual inspection as a poor indicator of the efficacy of the manual clean and has called for adjuncts in the cleaning process to decrease the environmental CFUs.^11,12^

Techniques used to potentially reduce environmental CFUs include laminar airflow, reducing traffic within the OR, surgical gowning with air outlets, and the use of ultraviolet (UV) lights.^5,13^ While other methods (e.g., laminar airflow) simply displace CFUs from the surgical site, techniques utilizing UV light inactivate microorganisms from irradiated areas.^3,5,6,13–15^ The inactivation of microbes occurs due to microbial cells absorbing UV-C photons causing critical damage to the genomic system preventing replication and survival.^16^ UV light is germicidal at specific wavelengths and has been shown to reduce infections hypothetically due to a reduction in CFU or the obtaining of ultraclean air (defined as a concentration of 10 m^−3^ or less airborne bacteria).^5,17^ However, some recent data has failed to support the clinical value of UV treatment programs.^18^ Although there is a consensus of support for the efficacy of UV light in lowering environmental pathogen transmission during surgical procedures, the potential short and long-term side effects (conjunctivitis, corneal injury, skin cancer, erythema, photokeratitis) of UV light to operative personnel has restricted its use in the operating theater during surgical cases.^5,6,19,20^

The most common UV lights used for germicidal purposes are low-pressure mercury (LPM) vapor arc lamp and xenon lamp technology which emit around 254 nm and broad UV spectrum respectively.^21^ These traditional types of UV lights can only be used in unoccupied spaces due to their health risks, but a newly emerging UV light source, the UV-C light-emitting diode (LED), has the potential to replace the traditional UV lights for disinfection purposes. UV-C LEDs typically emit at 265–275 nm and have a comparable or slightly better life than LPM, are directional emitters, are efficient, and do not contain hazardous material.^22,23^ The use of directional emission may reduce the impact of UV exposure on operative personnel, especially with a handheld device. The device is a “spotlight” disinfection and not whole room disinfection, changing the potential required safety features. As UV-C LEDs do not emit visual light, indicators such as blue LEDs can be used in commercial products to show where light is being directed. Additionally, other safety features like motion detection may be implemented with the device as well. Handheld UV-C LED has been shown to reduce CFU counts on previously infected surfaces when used in the optimal environment with direct beam exposure and a shorter target distance.^24^ Germicidal UV-C LEDs are currently used in the application of commercial water treatment,^25,26^ but there is a paucity of data regarding surface disinfection using this technology.^27,28^ A previous study showed promise in decreasing CFU near the operative field during TJA cases with a back table light utilizing the UV-C LED technology.^12^ Therefore, the purpose of this study was to assess the impact of a handheld UV-C LED disinfection device on the decontamination of HTS in the operating room (OR) when used as an adjunct to terminal cleaning. Our hypothesis was that UV-C LEDs would have a greater reduction in CFUs on HTS in the OR compared to manual cleaning alone.

## Methods

**Figure 1.**
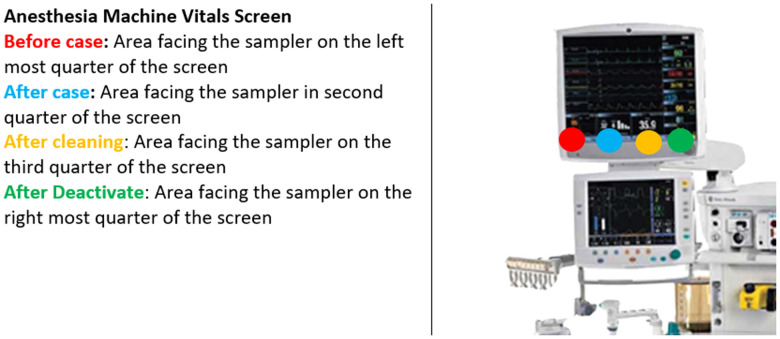
Visual location of samples collected from the anesthesia machine vitals screen.

**Figure 2.**
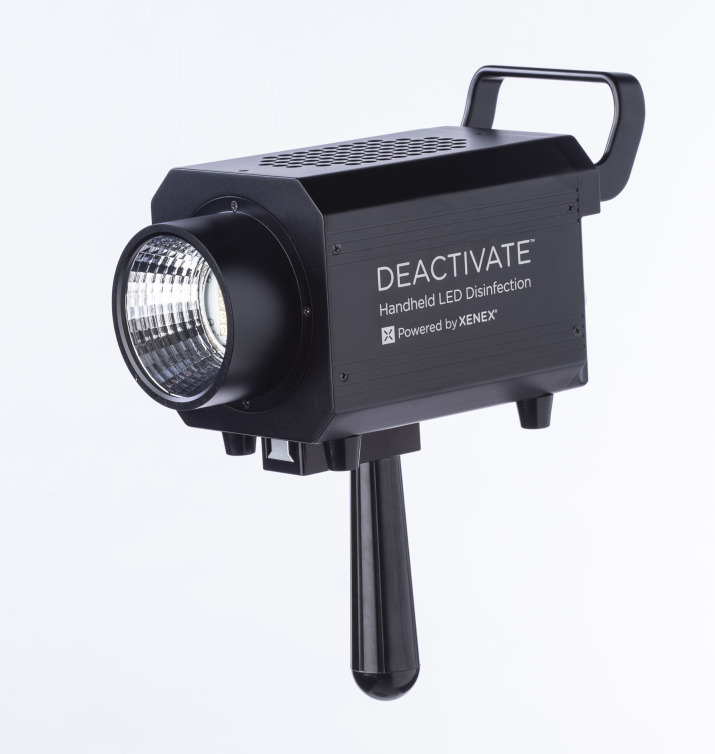
Handheld ultraviolet light-emitting diode device used for this study.

This study was approved by our institutional review board prior to initiation. The study was conducted between 03/02/2021 and 04/20/2021. This study was performed in a prospective manner. HTSs near the operative field that were the least likely to be thoroughly cleaned were identified from a previous study at our institution^8^ which included: anesthesia machine vitals screen, supply cabinet doors, nurse’s documentation station, electrocautery control unit, and the anesthesia cart table. These HTS had specifically designated predetermined areas for our tryptic soy agar (TSA) contact plates that were consistent throughout the study (Figure 1). These plates are used to determine the bacterial load on the selected HTS. The TSA plates were utilized before the initiation of the case and at the conclusion of each case. Next, data was obtained from the TSA *after* the manual clean was complete. The manual cleaning was done in the standard fashion and the perioperative team was unaware of the initiation or completion of this study. The manual clean at our institution is standardized and the same staff performs this process daily. Manual cleaning was performed with our standard solution (Prepzyme Forever Wet) and dwell times were in accordance with labeling requirements. After the manual clean and collection of data from the selected surfaces, a novel handheld UV-C LED light device was then immediately used over these selected surfaces as an adjunct to the manual disinfection (Figure 2). The handheld device had a target distance of 2.6 feet and was held approximately 1 meter from the surface for 2 minutes. The wavelength for this product is 265nm. The bacterial load over the selected areas was then analyzed with a TSA after the use of the handheld device. One surgical suite was chosen for this study. This process was completed for a total of 7 operative days. The ORs utilized were rooms designed specifically for and only have primary and revision TJA procedures routinely performed during the day. All TSA plates were taken and incubated for 48 hours at 36° +/–1° C. CFU were recorded 48 hours after incubation.

### Statistical analysis

The mean and ranges of CFUs were recorded. CFU counts for each arm were compared using negative binomial regression. Plates with confluent growth of >250 colonies were identified as too numerous to count (TNTC), and assigned a value of 250 colonies. Due to the non-parametric distribution of the CFU data, a Wilcoxon signed-rank test of matched pairs was used to determine whether samples taken on the same surface had statistically different levels of contamination. All statistical analyses were conducted using Stata 16.0 (College Station, TX).

## Results

A total of 300 TSA plates were analyzed. 72 and 96 plates had CFUs recorded at 48 hours. The remaining 132 plates did not have growth. Table 1 shows the breakdown by surfaces. The vitals screens were the least contaminated surface. The anesthesia cart increased in contamination after a case while the other surfaces decreased. There was not a statistically significant difference between average CFU per surface before and after the surgical case (14.1 vs 13.5 respectively, range: 0–200, *P* = 0.9397). Manual cleaning significantly reduced CFU count by 74% to 3.35 (range: 0–200, *P* = 0.0162). CFU count was further reduced from post-manual cleaning levels using UV-C LED light disinfection system by 92% to 0.28 (range: 0–4, *P* < 0.0001). Table 2 shows the % change in CFU count for the 48-hour data.

**Table 1.** Total surface colony forming unit (CFU) counts (48 hours)

Surface	Before Case 48 H	After Case 48 H	After Cleaning 48 H	After Deactivate 48 H
Anesthesia Cart Table	233	416	7	4
Anesthesia Machine Vitals Screen	1	9	22	0
Electrocautery Control Unit	215	219	15	4
Nurses Documentation Station	205	151	9	9
Supply Cabinet Door	404	220	12	4
TOTAL CFU counts	**1058**	**1015**	**65**	**21**

**Table 2.** Mean total colony forming unit (CFU) counts by operating room status at 48 hours

CFU Counts	Before Case	After Case	After Cleaning	After Deactivate
TOTAL	14.1	13.5	3.5	0.3
% change		–4%	–74%	–92%
total % change				–**98%**

## Discussion

Environmental transfer of pathogen to patient has been shown to be a common cause of PJI.^29^ Manual cleaning and disinfection of surfaces in the hospital have been shown in multiple studies to be subpar or not in accordance with hospital policy.^30,31^ The combined use of germicidal spectrum (200–320 nm) UV light and laminar airflow has been shown to decrease CFUs possibly decreasing PJIs.^3,32^ Our data showed a significant decrease in CFUs as an adjunct to a manual clean on HTS. To our knowledge, this is the first study to show the use of a novel simple handheld UV-C LED may be an effective way to decrease environmental contamination in the OR.

The first use of UV light in the operating room was at Duke University, which showed a substantial decrease in infection rates.^33^ Multiple subsequent studies found similar results, showing a decrease in CFU both locally at the operative site as well as peripherally around the room.^34–36^ Despite the efficacy shown by UV light to reduce CFUs, the use of UV light in the OR has been limited by restrictive guidelines to terminal cleaning at unoccupied times.^5^ The justification for the use of traditional UV light in the OR during occupancy is outweighed by the environmental risks to OR staff.^5,6,19,20^

As previously stated, a newly emerging UV light source, UV-C LED light, is currently being studied as a possible alternative to traditional UV light sources in the OR. We previously showed a decrease in CFUs with a back table light during operative cases when compared to a sham light.^37^ UV-C LED light has many advantages such as simplified system design, flexible form factor, no start-up or cool-down time, directional emission, longer life, and lower maintenance and cost.^22,23^ The results of this study supported the use of UV-C LED light in reducing CFUs on HTS in the OR with a substantial decrease in CFUs following manual cleaning, which has the potential to lead to a decrease in contamination. The purpose of this study was not to study the relationship between UV-C LED light and PJI, but prior studies have shown a relationship between CFUs in the environment and PJIs.^5^

UV-C LED light is currently used for the commercial treatment of water,^25,26^ but has been minimally studied for use as a surface disinfectant in the OR.^13,27,28^ Our study examined the efficacy of using UV-C LED light as an adjunct to disinfectant of HTS in the OR and found that UV-C LED light is more efficacious than manual cleaning alone. This handheld device could be used on targeted surfaces between cases without requiring the OR suite to be unoccupied and therefore not changing the length of the turnover times. This study did not examine the relationship between UV-C LED light and the potential risk to operative personnel or PJI. Therefore, our results should be considered experimental until further studies can be done to ensure no risk to operative personnel and show a reduction in PJI. While UV-C LED light has many advantages, the disadvantages such as manual aiming of the device, safety concerns if the room is occupied, smaller disinfection areas, and thermal management problems must be considered as the life and reliability decrease with increasing temperature.^23^ Additionally, this technology requires trained employees to utilize this in a proper fashion to ensure its efficacy.

Our study was not without limitations that should be acknowledged. This study design was to analyze the relationship between UV-C LED light and CFU reduction, not PJIs. However, as previously mentioned, PJI is potentially associated with an increase in CFUs.^5^ Future studies looking at UV-C LED light with and without other technologies (i.e. laminar airflow) as well as versus traditional UV light to assess if a true difference exists between technologies. This study also was designed to assess the reduction in CFU level using UV-C LED light after manual cleaning was already done. Our previously published study showed that UV-C LED light alone significantly reduced CFU level of a back table in an OR after TJA.^13^ Due to a restriction on the upper limit of CFU level, we may have underestimated the effect of manual cleaning leading to a minimized effect. Due to this technology being studied for future use in an occupied OR, future studies on the effect of UV irradiation on protective equipment worn by OR staff during surgery as well as instruments within the OR should be done prior to being utilized. Various factors may play a role in studying the microbial environment. Our results suggest that there were no significant differences in contamination level from before or after the case. This may be due to sampling technique and other variables not accounted for in this study. Additionally, there was variation in the portion of the evaluated surfaces that were not cleaned which may bias the magnitude of the apparent benefit of the UV-C LED treatment. In environmental microbial studies, a range of factors leads to variability. These factors could include the type of surface being sampled, level of proteinaceous material on the surface, and the behavior of the cleaning technicians. Environmental studies should use aggregate data to assess risk and benefit of interventions. Additionally, our surfaces tested were derived from other studies at our institution.^8^ Additional sampling on other HTS would be helpful for future research. Currently, this UV-C LED technology is not readily available like other sources of UV-C. However, they are becoming more available at a reasonable cost and have a wavelength range (265–275 nm) that makes them viable for healthcare settings. The ideal range with this technology for microbial activation needs to be determined in larger studies. This study was limited by sample size secondary to resources which certainly may have affected the results. The sample size we used was determined from previous studies on environmental bioburden sampling. Additionally, this study was performed in a total joint arthroplasty setting by one fellowship-trained surgeon. This may limit the generalizability of these findings. Hand hygiene compliance rates were not monitored during the study and certainly could have influenced the results of this study (i.e. contamination of HTS). Contamination was higher in the anesthesia vital screen after the case which may be affected by hand hygiene or could indicate cross contamination during the cleaning process. Samples were collected from the same area and this could have led to a bias in the reduction of microbes from double sampling. Lastly, future studies should also specify the recovered organisms to determine whether known pathogens are present in each treatment group. Despite these limitations, we feel this initial look at this technology shows promise for future studies and outcomes in TJA patients.

A handheld UV-C LED disinfection device decreased environmental contamination near the operative field in HTS areas. The results of this study show a positive prospect of the use of this technology as an alternative to traditional UV light in reducing CFUs but further studies are necessary to determine if this correlates with a decrease in PJI.
